# Legitimation Without Argumentation: An Empirical Discourse Analysis of ‘Validity as an Argument’ in Assessment

**DOI:** 10.5334/pme.1404

**Published:** 2024-10-03

**Authors:** Benjamin Kinnear, Daniel J. Schumacher, Lara Varpio, Erik W. Driessen, Abigail Konopasky

**Affiliations:** 1 Department of Pediatrics at University of Cincinnati College of Medicine in Cincinnati, OH, USA; 2 Department of Pediatrics at Cincinnati Children’s Hospital Medical Center/University of Cincinnati College of Medicine in Cincinnati, OH, USA; 3 Department of Pediatrics at the Perelman School of Medicine, University of Pennsylvania, USA; 4 Children’s Hospital of Philadelphia in Philadelphia, PA, USA; 5 School of Health Professions Education (SHE) at Faculty of Health at the Medicine and Life Sciences of Maastricht University in Maastricht, NL; 6 Geisel School of Medicine at Dartmouth in Hanover, New Hampshire, USA

## Abstract

**Introduction:**

Validity is frequently conceptualized in health professions education (HPE) assessment as an argument that supports the interpretation and uses of data. However, previous work has shown that many validity scholars believe argument and argumentation are relatively lacking in HPE. To better understand HPE’s discourse around argument and argumentation with regard to assessment validity, the authors explored the discourses present in published HPE manuscripts.

**Methods:**

The authors used a bricolage of critical discourse analysis approaches to understand how the language in influential peer reviewed manuscripts has shaped HPE’s understanding of validity arguments and argumentation. The authors used multiple search strategies to develop a final corpus of 39 manuscripts that were seen as influential in how validity arguments are conceptualized within HPE. An analytic framework drawing on prior research on Argumentation Theory was used to code manuscripts before developing themes relevant to the research question.

**Results:**

The authors found that the elaboration of argument and argumentation within HPE’s validity discourse is scant, with few components of Argumentation Theory (such as intended audience) existing within the discourse. The *validity as an argument* discourse was legitimized via authorization (reference to authority), rationalization (reference to institutionalized action), and mythopoesis (narrative building). This legitimation has cemented the *validity as an argument* discourse in HPE despite minimal exploration of what argument and argumentation are.

**Discussion:**

This study corroborates previous work showing the dearth of argument and argumentation present within HPE’s validity discourse. An opportunity exists to use Argumentation Theory in HPE to better develop validation practices that support use of argument.

## Introduction

Validity has long been a revered concept in health professions education (HPE). Demonstration of validity is often seen as imperative since, “without evidence of validity, assessments in medical education have little or no intrinsic meaning [1].” Despite its importance to HPE, assessment validity is conceptualized in multiple ways [2]. Some see validity as a property of an assessment instrument, with a given test or tool being labelled “validated” for use across differing contexts. Over the last two decades, HPE has drawn from other fields such as Psychology and Language Testing to frame validity as an argument that supports the interpretations and uses of assessment data [3,4]. In this orientation, validity is not an immutable property of an instrument, but a context-specific judgment about interpretations and uses of assessments. This argument-based conceptualization has gained traction in HPE in recent years, for a variety of possible reasons. HPE has growing epistemological diversity (i.e. diversity of theories regarding what is knowledge) [5] with subjectivity and constructionist approaches being increasingly embraced in assessment [6,7,8]. The importance of context in assessment has similarly gained recognition [9,10], making the idea that an assessment instrument can be “validated” in a context-independent way less aligned with broader discourses in HPE. Concurrently, programs are increasingly using complex, multifaceted programs of assessment [11] that integrate and synthesize multiple types of assessment data that may not be appropriate for traditional psychometric validation approaches. *Validity as an argument* is a conceptualization that allows for epistemological diversity, context specificity, and multiple assessment approaches.

However, the adoption of *validity as an argument* in HPE has occurred without development of what is meant by *argument*. Arguments exist to have real-world impacts such as advancing an idea, defending a viewpoint, or changing a policy [12]. The process of using an argument in the real world is called *argumentation*. By its very nature, argumentation (and, therefore, an argument) is social (i.e. exists between people or groups), invokes reason (i.e. uses rationality to some degree), and relates to a particular standpoint or opinion [12]. The concepts of *argument* and *argumentation* in the context of validity have been explored through Argumentation Theory, a theoretically and philosophically diverse field of study concerned with the development, execution, and evaluation of various forms of argument [12]. Each argumentation orientation found in Argumentation Theory brings assumptions and expectations. For example, Informal Logic is an orientation that emphasizes identifying and structuring arguments, with evaluation using normative standards such as relevance, sufficiency, and acceptability. In contrast, New Rhetoric is a different orientation that centers on audience values and norms rather than on a particular structure, with the goal of the argument aimed at persuading the audience, not fulfilling normative standards. There are many orientations within Argumentation Theory, each drawing on different aspects of scholarly discourse and each leading to different approaches to argument development, organization, operationalization, analysis, and evaluation.

Validity arguments in HPE rarely (if ever) explicitly attend to the argumentation orientation undergirding validation work [13]. In our interview study of HPE experts regarding their conceptualizations of argument and argumentation related to assessment validity, participants described an absence of explicit reference to argumentation in HPE validation work. Specifically, they noted a shortage of attention to argument audiences, evaluation, and situated context. Participants perceived that peer-reviewed publications (often from North America or Europe) are used to authoritatively justify validity approaches. Trying to create or evaluate arguments without an explicit argumentation orientation is like chess players who have the game pieces but lack the rules. If *validity as an argument* is to continue to be used in real-world HPE assessment work, our field requires a better understanding of the argument and argumentation *discourses* (i.e., “way[s] of signifying a particular domain of social practice from a particular perspective”) [14] that exist within peer reviewed publications. Therefore, in this study, we used discourse analysis to examine how argument and argumentation are manifested in HPE publications around validity. By developing this understanding, we hope to uncover implicit argumentation concepts, and to better understand the discourses driving *validity as an argument* in peer reviewed publications.

## Methods

### Approach

Our work is undergirded by Norman Fairclough’s approach to discourse analysis, which holds that “one cannot properly analyse content without simultaneously analysing form.” [14]. In other words, to understand ways of signifying (i.e., discourses), one must look at the *language*, i.e., at the *signifiers*. Yet, unlike Fairclough, we do not take “discourse” as a social process to be fully deterministic. Instead, we follow Archer in seeing human reflexivity as mediating the forces of structure and agency [15]. We then draw on Fairclough’s approach alongside other tools offered by Gee [16] and Laclau and Mouffe [17] to engage in methodological *bricolage*, using multiple methods of analysis to understand validity in HPE as “part of a historically situated complex system [18].”

We began with the research question, “What aspects of argumentation implicitly or explicitly manifest in influential HPE validity manuscripts published in peer-reviewed journals?”. Our goal was not triangulation with our previous HPE expert interviews [13], but rather to seek a richer understanding of how *argument* and *argumentation* manifest and are shaped by the language used in HPE validity publications. To achieve this goal, we relied on discourse analysis, which allows researchers to understand how language influences and is connected with social practices [19,20]. Since peer-reviewed publications are a major conduit of discourse within HPE, we used critical discourse analysis to understand how the language in influential manuscripts reflects discourses of arguments and argumentation relating to validity.

### Document sampling strategy

The goal of this study was not to conduct a comprehensive discourse analysis on all manuscripts addressing validity in HPE; instead, we sought to compile a corpus of manuscripts that directly addressed discourses on validity arguments within HPE. To that end, we used multiple search strategies to identify candidate manuscripts. First, one author (BK) met with an academic librarian to formulate search strategies to capture as many HPE articles on validity and validation as possible. We chose to only search in PubMed because our research question focused on HPE’s discourse, and most HPE journals are indexed within PubMed. Nine different search strings were used (Appendix A), resulting in 800 unique manuscripts. BK reviewed the titles of these manuscripts to identify papers that promised to explore validity or validation. BK read the abstracts of the promising manuscripts to identify papers that explored the philosophical, theoretical, or practical aspects of validity arguments, even if this exploration was only in part of the manuscript (e.g. introduction). Manuscripts focusing on assessment more broadly without rich discussion on validity arguments were excluded. If a manuscript was felt to be useful in answering the research question, it was added to the corpus for full text analysis. To ensure that we had not missed any particularly relevant publications, we contacted the authors of a previous discourse analysis on validity in HPE [2] and obtained their corpus, reviewing it for manuscripts that would contribute to answering our research question. We also mined the references of the sampled publications looking for manuscripts relevant to our research question that had yet to be identified. We limited inclusion to manuscripts that were within the HPE literature.

BK completed an in-depth review of 59 manuscripts after the above process, searching for papers that provided a substantive discussion of the conceptualization or operationalization of validity arguments in HPE. This resulted in 38 articles being selected for analysis. All articles that were excluded by BK were reviewed by a second study author (AK) to ensure that their exclusion would not remove data that would be meaningful in answering our research question. This second author’s (AK) screening led to the re-inclusion of one article. Our final corpus was 39 manuscripts spanning in publication date from 2003 to 2022. Table 1 provides a summary of the final corpus, with more details available in Appendix B.

**Table 1:** Summary of the included manuscripts.


Publishing journal	Medical Education (n = 13)

	Advances in Health Sciences Education (n = 7)

	Academic Medicine (n = 2)

	Medical Teacher (n = 2)

	Advances in Medical Education and Practice (n = 1)

	Advances in Simulation (n = 1)

	American Journal of Medicine (n = 1)

	American Journal of Pharmaceutical Education (n = 1)

	American Journal of Surgery (n = 1)

	Current Urology Reports (n = 1)

	Currents in Pharmacy Teaching and Learning (n = 1)

	Internal Medicine Journal (n = 1)

	Journal of Graduate Medical Education (n = 1)

	Journal of Clinical Nursing (n = 1)

	Journal of Endourology (n = 1)

	Journal of Pediatrics (n = 1)

	MedEdPortal (n = 1)

	Perspectives on Medical Education (n = 1)

	Surgical Endoscopy (n = 1)

Country of first author professional affiliation	United States (n = 22)

	Canada (n = 9)

	Australia (n = 2)

	United Kingdom (n = 2)

	Egypt (n = 1)

	Germany (n = 1)

	Netherlands (n = 1)

	New Zealand (n = 1)

	Sudan (n = 1)


Note that one author listed affiliations in two countries, both of which are listed.

### Analysis

Our bricolage approach to discourse analysis drew on multiple traditions. First, like Laclau and Mouffe [17], we approached discourse as a net of linguistic terms and processes through which meaning is constantly created and negotiated [17]. In particular, based on how we saw terminology being used in our prior work [13], Laclau and Mouffe’s concept of nodal points (i.e. words/concepts around which other concepts are ordered) helped us to consider the terms *validity* and *argument* as knots in the net of HPE’s discourse; these words, as nodal points, derive their meaning by being situated in particular positions in HPE’s ever shifting discourse. By examining how these terms are used in our corpus as nodal points, we were able to understand how these words were prioritized and used to set meaning within HPE’s discourse.

Our approach was also informed by Fairclough’s critical discourse analysis [21] which frames discourse as a social practice “that both reproduces and changes knowledge” [17]. It emphasizes that individual linguistic choices (i.e., words) “can only be understood in relation to webs of other texts and in relation to the social context” [17]. We used the concept of *legitimation* (i.e. the process by which systems of authority seek to establish a belief as legitimate) to explore how discourses related to *validity as an argument* are established and empowered. This bricolage approach employed multiple tools to understand what the authors of each manuscript in the corpus meant or intended to accomplish with their use of language.

Guided by what we found in our previous work on Argumentation Theory [22], we developed an analytic framework—acting as bricoleurs to bring together Laclau and Mouffe, Fairclough, and Gee —consisting of questions that we asked of the data during coding (Table 2). Each question served to apply various tools from Gee to explore the language used in our discourses of interest. One author (BK) read each of the manuscripts in the final corpus to familiarize himself with the texts. He then reread and coded four manuscripts using the analytic framework, followed by a second co-investigator (DS, LV, AK, or ED) independently coding one of the four manuscripts each. We then met to review our coding and to discuss the fit of our analytic framework to our research aim of elucidating how authors used language to set meaning and influence HPE’s understanding of validity arguments. After this meeting, BK coded 16 more manuscripts, assigning a second coder for each (every co-investigator coded 5 of the 20 initial manuscripts). The team met again to discuss codes, language patterns, nodal points, and developing themes. At this meeting, the team further developed a shared mental model for future coding and theme development. BK then read and coded the remaining 18 manuscripts using the established model. We felt that we had reached theoretical sufficiency prior to coding all 39 manuscripts; however, we analyzed the entire corpus to identify rich quotes or examples that might otherwise be missed. We used Quirkos (Quirkos 2.5.2 Computer Software, 2022) to facilitate primary coding, to develop a visual representation of how codes could be grouped and related, and to facilitate reflections and interpretations throughout the process.

**Table 2:** Analytic framework used by the authors for primary coding.


QUESTION	GEE’S DISCOURSE ANALYSIS TOOLS [16]	RATIONALE FOR ASKING

How are audiences of validity arguments addressed?	**Fill in tool** – asking what is not being explicitly said but is inferable **Making strange tool** – acting as an outsider in the discourse	Audience was identified as central in validation in a previous study

What evaluation criteria is explicitly stated or implied by the authors?	**Fill in tool** – asking what is not being explicitly said but is inferable **Making strange tool** – acting as an outsider in the discourse	*Logic* = truth between premises/conclusions *Informal logic* = relevance, sufficiency, acceptability *New Rhetoric* = convincing, persuasion *Pragmadialectics* = winning against an interlocutor

Who is in the author group? What authority is claimed or implied for their writing of the topic?	**Identities building tool** – asking which social identities are assumed or enacted **Significance building tool** – asking how language builds or diminishes significance	These papers set the discourse for our field. Writing them is an implicit claim to authority.

What argumentation ideas/concepts/terms (e.g. claim, warrant, rebuttal) are explicitly used by authors? What might this imply?	**Fill in tool** – asking what is not being explicitly said but is inferable **Making strange tool** – acting as an outsider in the discourse	This may help understand how much argumentation is emphasized or used in HPE validity.

What argumentation concepts are being assumed by authors?	**Fill in tool** – asking what is not being explicitly said but is inferable **Making strange tool** – acting as an outsider in the discourse	Much about argumentation is not fully explored in HPE validity writing.

What is being problematized about argumentation? Who is addressed as having power to ‘fix’ the outlined problem?	**Activities building tool** – asking what activity a discourse is seeking to perform **Identities building tool** – asking which social identities are assumed or enacted	A previous study suggested argumentation is not really happening in HPE. (ref 13) What, if anything about arguments/argumentation has already been problematized?

How do the authors use intertextuality? How much dialogicity do they use (i.e. description vs naming vs quotes). What references are made or what sources of knowledge are employed?	**Intertextuality tool** – Asking how a discourse draws from other texts	The names of validity scholars (e.g. Messick, Kane, Cook) are used frequently in published manuscripts. Why do authors use intertextuality that way?

What are the “nodal points” (privileged sign around which other signs are ordered) that help set meaning related to argumentation? How are they used?	**Significance building tool** – asking how language builds or diminishes significance	Certain terms, concepts, or ideas may be important in describing/defining arguments/argumentation


### Reflexivity

Our team members’ unique areas of expertise and personal backgrounds informed our data analysis and interpretation. AK is a critical qualitative researcher with a background in linguistics. She came to this work with a particular interest and expertise in authors’ discursive techniques. ED, LV, DS, and BK brought social constructionist views to the work, viewing knowledge as being constructed by and between people. DS and BK are clinician educators who actively engage in HPE research. BK is a PhD candidate with expertise in programmatic assessment whose interpretations were influenced by prior research in the area of validity argumentation [13,22], and by his experience as a frontline clinician educator and residency program leader. DS is a pediatric emergency medicine physician and PhD-trained research scientist with expertise in competency-based medical education (CBME) and programmatic assessment. ED is an educational scientist whose PhD research included examining the validity of portfolio assessment. His experience as editor-in-chief of a HPE journal has influenced his view of the discourses created through the publication process. LV is a PhD-trained HPE researcher with expertise in argumentation theory, rhetoric, and professional communication, and in qualitative research’s paradigms, methodologies, and methods. This expertise shaped her interpretations of the manuscripts.

As a team, our varied educational backgrounds, areas of research expertise, and work experiences in HPE meant that we often pushed each other’s interpretations and questioned the various kinds of legitimacy that were being harnessed in any given manuscript. Also, given that this project was undertaken by BK as part of his thesis research and that the other members of the team included supervisors of his thesis research and collaborators invited to the project because of their expertise, the team was sensitive to the power dynamics shaping the collaboration. The team focused on simultaneously meeting two goals: to support BK in developing research skills by having him lead the analysis of the corpus; and to ensure that rigorous, theory informed discourse analysis was carried out.

## Results

Although *validity as an argument* is a prominent discourse within HPE literature (as noted previously by St. Onge et al) [2], we found the elaboration of *argument* and *argumentation* within this discourse to be scant. Specifically, we found that the discourse of validity arguments neglected or minimized audiences for those arguments. The discourse was legitimated via multiple methods that cemented *validity as an argument* in HPE despite minimal exploration of what argument and argumentation are. Below, we elaborate on four aspects of the discourse on HPE validity arguments: lack of argumentation, absence of audience, epistemological tension, and legitimation.

### Validity Arguments Without Argumentation

Relatively few manuscripts explained the concepts of argument or argumentation. In nearly all papers, the *validity as an argument* discourse centered around the concepts of *evidence* and *inference*. These concepts served as nodal points when describing validity as an argument and were often linked to two specific frameworks: evidence was often described within Messick’s framework [23] and inference was often used in the context of Kane’s framework [24]. Most articles focused significantly on evidence and did not describe other aspects of argument or argumentation. Multiple articles did not describe nor provide detailed explanations on arguments at all. Rarely, articles explicitly noted that validity requires more than evidence collection. For example, one author noted: “Contrary to popular opinion, evidence does not speak for itself. To convince a jury, evidence must be selected, interpreted and presented in a carefully orchestrated argument [4].” Despite the prevalence of the *validity as argument* discourse, an approach to building an argument was rarely articulated.

No explicit articulation of an argumentation theory, orientation, or philosophy (e.g. Informal Logic, New Rhetoric) appeared anywhere in our corpus. Perhaps as a corollary, papers in our corpus used general and heterogenous language when describing criteria for evaluating validity arguments such as “coherent” [11,25,26,27,28,29,30,31] “credible” [26,32,33], “clear” [11,31], “complete” [11], defensible [28,30,32,33,34,35], plausible [3,11,25,31], justifiable [4,34,36,37,38,39], well-grounded [4,36], trustworthy [33,36,40], reasonable [1,33,36], sound [32,41], accurate [39,40], sufficient [2,29,32,40,42], acceptable [35,43], and specific [11]. One author explicitly acknowledged the difficulty in elaborating evaluation criteria for validity arguments, writing: “In theory it is difficult to define the point at which we can claim that a test is valid.” [44] Across the corpus, there was considerable ambiguity about how validity arguments should be developed and evaluated.

### Arguments Without Audience

Similarly, the *validity as an argument* discourse did not describe who can or should be the audiences addressed by such arguments. By *audience* we mean the person, group, or institution who receives, interprets, and evaluates validity arguments. The term audience only appeared one time within the entire corpus in reference to validity arguments. While this manuscript did not explicitly state who the audience is or should be, it acknowledged the centrality of audience values in determining validity of arguments: “[an argument] is not ‘valid’ or ‘invalid’, but varies by degrees and will be more or less convincing to different audiences.” [40] Some manuscripts used terms such as users [26,29,45] or stakeholders [32,35,44], but these terms were often subordinate to the nodal points of evidence and inference. One author made explicit that readers of journal articles are the target audience, writing “it is up to the reader to evaluate whether sufficient evidence is provided to support the adaptation of a tool [46].”

Several manuscripts did not address audience at all; instead, they focused entirely on evidence. In fact, a common manuscript organization strategy was to have subsections focused entirely on evidence types or sources without attending to the broader argument or audience. Some manuscripts intimated the existence of an audience through language that implied an exchange of information such as “argue for and against” [1], “theory, hypotheses, and logic which are presented” [1], “no validation work can possibly address all concerns” [46]. However, these manuscripts gave no clear description to whom the arguments are argued or presented, nor whose concerns should be addressed. The minimization (or absence) of audience reduced the engagement of social exchanges as part of HPE validity arguments.

### Epistemological Tensions in Validity Discourse

The language used to describe nodal points revealed a diversity of epistemological worldviews within the *validity as an argument* discourse. By *epistemology* we mean implicit beliefs about how knowledge is generated or used [5], in this case specifically regarding validity arguments. For example, the nodal point of evidence was frequently described as being “obtained” [37], “compiled” [28], “gathered” [39], “sought” [33,39], or “collected” [4,26,27,33,47], implying that evidence objectively exists and, like a buried fossil, waits to be discovered. In contrast, arguments were frequently described as being built [11,34], formulated [29], or constructed [26], implying that they rely on human agency and decisions to exist. These characterizations often co-occurred in the same text or even within the same sentence: “The process of building an argument and collecting evidence supporting that interpretation and its decision-making conclusions, with each different learning assessment use and among different test-takers, is termed validation [28].”

These descriptions imply an objective, more post-positivist view of *evidence* with a more subjective, interpretivist conceptualization of *arguments*. Such implicit views were reflected in how evidence and argument were seen to be operationalized in validity. Authors described “closing gaps” [4,27] in evidence, again implying an objective and ideal type of evidence that researchers should strive to uncover. One author used terms that are often used in qualitative research but have a postpositivist underpinning [48], such as “triangulate” [32] and “saturation” [32], in describing the collection of validity evidence. Another author highlighted the interpretive nature of arguments, noting: “Kane makes no claims about representing a ‘truth’, and instead argues for a justified belief obtained using whatever means necessary, leaving truth in the background [38].” In sum, the language used within HPE’s *validity as argument* discourse contains heterogeneous epistemological groundings that can be contradictory across manuscripts and even within a single manuscript.

### Legitimating Validity as an Argument

Legitimation is the use of textual explanations and justifications for defending or upholding how things are done [21]. Authors in our corpus used multiple methods of legitimation to cement the *validity as an argument* discourse. Nearly every text used authorization (i.e. reference to authority) via intertextuality (i.e. relating different texts to one another). While referencing previous literature is part of scholarly writing’s tradition, authors in our corpus nearly always referenced high-profile validity scholars by name. While some scholars were named multiple times (e.g. Cook, Cronbach, Downing), Messick and Kane appeared in almost every paper, sometimes appearing with direct quotes from their original work. This demonstrates a high degree of intertextuality for purposes of authorization. High intertextuality includes directly naming or quoting other authors [21]. One author in our corpus even portrayed Kane as speaking directly with readers using the present tense, writing “Kane reminds us…” [34] In addition to citing specific scholars, authors in our corpus also used high intertextuality in referencing the Standards for Educational and Psychological Testing [49] as a method of authorization: “All validity is construct validity in this current framework, described most eloquently by Messick and embodied in the current Standards of Educational and Psychological Measurement” [1]. By referencing Messick and Kane by name and frequently, and by citing authoritative standards, the manuscripts in the corpus create the impression that an accepted consensus exists around the *validity as an argument* discourse which bolstered the discourse’s legitimacy, despite the fact that no such consensus exists.

Another legitimation approach we observed in the corpus was mythopoesis, which conveys legitimacy through narrative [21]. Numerous papers in our corpus included a historical narrative about how the conceptualization of validity has changed over time [1,4,25,26,28,29,30,34,36,37,44,45,50,51]. There were striking similarities in the narratives, often beginning with validity’s conceptualization in the 1950s and invoking the same validity scholars’ names (e.g.., Messick and Kane) as characters in the narrative. Multiple papers described how validity has “evolved” over time [25,26,30,33,37,52,53]. The term *evolve* implies that validity changed into an improved, more advanced state, thereby lending legitimacy to the current argument-based conceptualization. This narrative often framed argument-based approaches as “contemporary” [1,32,33,35,36,37,41,43,45] or “modern” [2,32,34,35,45,50,52], while non-argument based approaches were labelled as “outdated” [28,35,37,45], “misconceptions and malpractices” [37], and “antiquated” [2,54]. The evolutionary narrative was often presented with language that implied how the world ought to be such as “should” [27,30,32,34,35,36,39,40,53,55,56] or “must” [1,11,32,41,47,57]; implied a request or command such as “Provide information such as who created the instrument” [57]; or gave explicit recommendations about how validity ought to be conceptualized or operationalized, such as “Recommendation 1: Use current validity, reliability, and validation language that reflects today’s understanding of these foundational concepts” [34]. The narratives in HPE’s validity discourse (e.g. modern vs outdated, evolution, should/must, commands, recommendations) further reduced the possibility of questioning the *validity as an argument* discourse.

Rationalization, which invokes societal or institutional knowledge [21], is a final legitimation approach we found in the corpus. The language used to describe aspects of validity arguments often echoed the scientific method—a stalwart of knowledge and progress. For example, multiple authors used terms like “hypothesis” [1,25,29,30,33,36,37,45] and “theory” [3,11,25,33,34,35,58] in describing validity and validation, grounding the discourse in traditional, accepted scientific language. One author wrote: “Validity refers to the impartial, scientific collection of data, from multiple sources, to provide more or less support for the validity hypothesis and relates to logical arguments, based on theory and data, which are formed to assign meaningful interpretations to assessment data [1].” This quote borrows language found in scientific, empirical papers, and so reinforces the legitimacy of the *validity as an argument* discourse. This use of terminology legitimizes validity by entrenching it in the language of science.

## Discussion

This study builds on our previous work in which experts explained that argument and argumentation are largely absent in HPE validation work [13]. Our analysis highlights how the *validity as an argument* discourse was dominated by *evidence* without significant attention paid to the argumentation principles that might tie such evidence together. This study also corroborates previous findings [13] in which validity experts stated that audience, a key component of argument, is often minimized or ignored entirely in HPE validity. Most argumentation orientations explicitly debate the identities, roles, and centrality (or lack of centrality) of audiences for arguments [12]. Some argumentation orientations minimize audience importance (e.g. Logic) [59,60] while others entirely center on audience values and needs (e.g. New Rhetoric) [61,62]. In HPE, this debate about the role of audience to influence validity’s argument has yet to occur in a meaningful way. Some manuscripts in this study’s corpus briefly referenced or indirectly implied the existence of an audience, but none deeply explored how audiences should interact with or evaluate validity arguments, nor how much a specific audience’s views and values should guide validation work. Our findings do not suggest the presence of conflicting views on audience in HPE validation, but rather an absence of views. Audience seems to have been left behind in the immigration of argument into HPE validity. Ignoring or minimizing audience neuters the power of argument, transforming it simply into a validity claim. As argumentation theorist Frans van Eemeren wrote, “By itself, holding an opinion is not enough to initiate argumentation. Arguing makes sense only if there is a listener or reader who entertains doubt about an opinion or has a diverging opinion” [12]. In order to move from validity opinions to validity arguments, HPE must better define the role and identity of its audiences.

Our corpus was comprised of published manuscripts, and one might argue that reviewers, editors, or readers are serving as audiences. However, given the contextual and situated nature of validity [13], those audiences are unlikely to be meaningful stakeholders for most published validation work. Journal reviewers and editors are meaningful and necessary audiences for knowledge advancement, but the argumentation mechanisms used in HPE validation should allow for the identification of and engagement with context-relevant and -specific audiences. For that to happen, audiences need to be identified and engaged in co-creating validity arguments. Co-creation might include having validity committees that meet to review and discuss validity arguments, asynchronous sharing and evaluation of validity arguments between argument developers and stakeholders, or other approaches that best engage intended audiences. Given the context-dependent nature of validity arguments [2,29,37], as well as the dearth of diversity in the HPE journal editorial world [63], actual stakeholders and end users impacted by assessment decisions (e.g. learners, local program leaders, patients) should be highly sought after audiences. Additionally, diverse audiences that represent multiple backgrounds and experiences should be prioritized to ensure equitable arguments are being made. By deliberately attending to the function of audience, perhaps HPE validity can more tangibly meet its social imperative to serve learners and patients [32].

Our analysis found that published HPE manuscripts use multiple approaches to legitimate the discourse of *validity as an argument*. While we cannot say for certain why legitimation is so frequently used, we have several hypotheses. Legitimation may simply be part of the natural process of translating new ways of thinking across disciplines. The *validity as an argument* discourse did not originate in HPE, but, like many concepts, it immigrated [64] to HPE from fields such as Measurement and Language Testing. Authors may be using legitimation as a rhetorical strategy to promote uptake and integration of new ideas into HPE. Legitimation may also be an indirect way to offset the lack of philosophical and theoretical grounding of argument and argumentation in HPE’s validity discourse. Such legitimation could entrench *validity as argument* as the authoritative discourse in HPE without having to fully reconcile aspects of argument or argumentation that may or may not fit with current practices. Our previous work suggested that HPE has largely adopted *validity as an argument* without fully integrating theories or practices used in other forms of argumentation [13,22]. Legitimation may be a form of compensation—in other words, legitimation may uphold the conceptualization of *validity as argument* despite the fact that we are unsure of what that actually means.

Rather than a weakness, we see the lack of argument and argumentation in HPE as an opportunity. HPE can pull from the rich history of Argumentation Theory to answer questions such as: By which qualities should validity arguments be evaluated and using what threshold? What argument structures might best facilitate evaluation? How should audience values impact argument content? Should specific or general/universal audiences be prioritized? With regard to argument evaluation, the language used in HPE’s discourse implies multiple different argumentation orientations such as Informal Logic (e.g. sufficient, acceptable, defensible) or New Rhetoric (e.g. plausible, convincing). Clarifying which argumentation orientation is being used helps set the ground rules for what criteria are used to evaluate validity. In other words, more explicit argumentation expectations would provide the ‘rules of chess’ to those who hold the game pieces. Exploring such questions may also help ease epistemological tensions. We are not advocating for any particular worldview, and indeed believe that the multiple epistemologies used in HPE enrich our field [65]. Rather, we hope that better articulated argumentation orientations could improve compatibility [38] between philosophical worldviews and argumentation approaches being used.

Our study has multiple limitations. First, we began with the presumption that *validity as an argument* is the predominant view of validity in HPE. We recognize that other views on the nature of validity exist concomitantly in our field [2]. However, we believe that *validity as an argument* is currently the most widely used conceptualization. Second, we only analyzed a sample of the HPE validity literature; we do not offer an interpretation that represents every published manuscript. Our focused approach is reflective of our critical and social constructionist worldviews in which our goal was not to seek an objective truth, but to develop understanding informed by our experiences and expertise. Third, we did not include specific validation studies (e.g. those of a specific assessment instrument), instead focusing on manuscripts that we felt were intended to influence our field’s views of validity and validation. Different discourses may exist in those studies that could lead to different results.

## Conclusion

Argumentation is missing from HPE’s discourse on validity arguments, fostering ambiguity in terms of who the audiences are (or should be) and how arguments are evaluated (or should be). More explicit use of Argumentation Theory in HPE, including the exploration of which orientations (e.g. Informal Logic, New Rhetoric) best support validity argumentation, may deepen our field’s understanding of how *validity as an argument* can best serve the interests of our learners, programs, and patients. Despite a lack of argumentation and minimization of argument (in favor of evidence), *validity as an argument* is legitimated via multiple mechanisms which serve to entrench it as a definitive conceptualization throughout HPE publications. Future research should include the use of critical theory to explore who this legitimation favors, and who it marginalizes or excludes.

## Additional Files

The additional files for this article can be found as follows:

10.5334/pme.1404.s1 Appendix A. Search strings used which resulted in 800 unique manuscripts for initial screening.

10.5334/pme.1404.s2 Appendix B. Full table of analyzed manuscripts.
