# Supplementary material for: Legitimation Without Argumentation: An Empirical Discourse Analysis of ‘Validity as an Argument’ in Assessment
Source: Perspect Med Educ. 2024 Oct 3;13(1):469–80. doi: 10.5334/pme.1404 (PMC11451546; doi:10.5334/pme.1404)
Supplement: Appendix A. — Search strings used which resulted in 800 unique manuscripts for initial screening. [file pme-13-1-1404-s1.pdf]

Appendix A: Search strings used which resulted in 800 unique manuscripts for initial screening

Search 1: 26 results

"Education, Medical"[Mesh] AND (("Educational Measurement/methods"[MeSH]) OR "Educational Measurement/standards"[MeSH]) AND valid*[Title] AND 1995/01/01:3000/12/31[Date - Publication] AND ("Editorial"[Publication Type] OR "Review"[Publication Type] OR "Consensus"[MeSH Terms] OR ("Consensus"[Title] OR "opinion"[Title] OR "commentar*"[Title] OR "perspective*"[Title] OR "discourse"[Title] OR "conversation"[Title]))

Search 2: 55 results (44 unique results)

"medical education"[Title/Abstract:~3] AND valid*[Title] AND 1995/01/01:3000/12/31[Date - Publication] AND ("Editorial"[Publication Type] OR "Review"[Publication Type] OR "Consensus"[MeSH Terms] OR ("Consensus"[Title] OR "opinion"[Title] OR "commentar*"[Title] OR "perspective*"[Title] OR "discourse"[Title] OR "conversation"[Title]))

Search 3: 159 results (145 unique results)

("Education, Medical"[Mesh] AND (assessment*[Title/Abstract] AND "validity"[All Fields]) AND 1995/01/01:3000/12/31[Date - Publication] AND ("Editorial"[Publication Type] OR "Review"[Publication Type] OR "Consensus"[MeSH Terms] OR ("Consensus"[Title] OR "opinion"[Title] OR "commentar*"[Title] OR "perspective*"[Title] OR "discourse"[Title] OR "conversation"[Title]))) NOT ("validation study"[Title/Abstract:~2] OR "validation tools"[Title/Abstract:~2] OR "assessment tools"[Title/Abstract:~2]))

Search 4: 228 results (142 unique results)

(((valid*[Title] AND (“Education, Medical”[Mesh] OR “medical education”[All Fields]) OR ((“assessments educational”[Title/Abstract:~3] OR “assessment educational”[Title/Abstract:~3] OR “assessments education”[Title/Abstract:~3] OR “assessment education”[Title/Abstract:~3]) AND valid*[All Fields])) AND 1995/01/01:3000/12/31[Date - Publication] AND ("Editorial"[Publication Type] OR "Review"[Publication Type] OR "Consensus"[MeSH Terms] OR ("Consensus"[Title] OR "opinion"[Title] OR "commentar*"[Title] OR "perspective*"[Title] OR "discourse"[Title] OR "conversation"[Title]))) NOT ("validation study"[Title/Abstract:~2] OR "validation tools"[Title/Abstract:~2] OR "assessment tools"[Title/Abstract:~2]))

Search 5: 120 results (111 unique results)

"valid*"[Title] AND "assessment*"[Title] AND ("Med Teach"[Journal] OR "Med Educ"[Journal] OR "J Grad Med Educ"[Journal] OR "BMC Medical Education"[Journal] OR "Adv Health Sci Educ Theory Pract"[Journal] OR "Perspectives on medical education"[Journal] OR "The American Journal of Medicine"[Journal] OR "The New England journal of medicine"[Journal] OR "Med Teach"[Journal] OR "Canadian medical education journal"[Journal] OR "JAMA"[Journal] OR "Adv Health Sci Educ Theory Pract"[Journal]) AND 1995/01/01:3000/12/31[Date - Publication]

Search 6: 57 results (46 unique results)

(“validity argument*”[Title] OR “validity evidence”[Title] OR “validity framework”[Title] OR “validity map”[Title]) AND (“medical education”[Title/Abstract:~4]) AND 1995/01/01:3000/12/31[Date - Publication]

Search 7: 190 results (103 unique results)

(“Educational Measurement”[MeSH] OR “Clinical Competence”[MeSH]) AND (valid[Title/Abstract] OR validation[Title/Abstract] OR validity[Title/Abstract]) AND “medical education”[Title/Abstract:~2] AND 1995/01/01:3000/12/31[Date - Publication] AND ("Editorial"[Publication Type] OR "Review"[Publication Type] OR "Consensus"[MeSH Terms] OR ("Consensus"[Title] OR "opinion"[Title] OR "commentar*"[Title] OR "perspective*"[Title] OR "discourse"[Title] OR "conversation"[Title]))

Search 8: 82 results (63 unique results)

("educational assessment"[Title/Abstract:~3] OR "education assessment"[Title/Abstract:~3]) AND "Reproducibility of Results"[MeSH Terms] AND "valid*"[All Fields] AND ("students, medical"[MeSH Terms] OR "education, medical"[MeSH Terms]) AND 1995/01/01:3000/12/31[Date - Publication]

Search 9: 158 results (120 unique results)

(assess*[Title] AND (“clinical competenc*”[Title] OR “medical education”[Title])) AND “valid*”[All Fields] AND 1995/01/01:3000/12/31[Date - Publication]
