# Supplementary material for: Legitimation Without Argumentation: An Empirical Discourse Analysis of ‘Validity as an Argument’ in Assessment
Source: Perspect Med Educ. 2024 Oct 3;13(1):469–80. doi: 10.5334/pme.1404 (PMC11451546; doi:10.5334/pme.1404)
Supplement: Appendix B. — Full table of analyzed manuscripts. [file pme-13-1-1404-s2.pdf]

Appendix B: Full table of analyzed manuscripts

Title	Lead Author	Journal	Year	Country of first author's professional affiliation	Article type
Validity: on the meaningful interpretation of assessment data	Downing	Medical Education	2003	USA	The Metric of Medical Education
Fundamental principles of validation, and reliability: rigorous science for the assessment of surgical education and training	Gallagher	Surgical Endoscopy	2003	USA	Original article
Validity threats: overcoming interference with proposed interpretations of assessment data	Downing	Medical Education	2004	USA	The Metric of Medical Education
Face validity of assessments: faith-based interpretations or evidence-based science?	Downing	Medical Education	2005	USA	Commentary
Current Concepts in Validity and Reliability for Psychometric Instruments: Theory and Application	Cook	American Journal of Medicine	2006	USA	Review
Assessment of clinical performance: gathering evidence	Wilkinson	Internal Medicine Journal	2007	New Zealand	Review
Conceptualising and classifying validity evidence for simulation	Andreatta	Medical Education	2009	Canada	Review
Reliability and Validity in a nutshell	Bannigan	Journal of Clinical Nursing	2009	United Kingdom	Methodological paper

A primer on the validity of assessment instruments	Sullivan	JGME	2011	USA	Editorial
Validity considerations in the assessment of professionalism	Clauser	Advances in Health Sciences Education	2012	USA	Reflections
From test validity to construct validity. . . And back?	Colliver	Medical Education	2012	USA	Review
Programmatic assessment and Kane's validity perspective	Schuwirth	Medical Education	2012	Australia	General article
Validity in WBA: expanding our horizons	Govaerts	Medical Education	2013	Netherlands	Cross-cutting edge
Educational Testing and Validity of Conclusions in the Scholarship of Teaching and Learning	Peeters	American Journal of Pharmaceutical Education	2013	USA	Special article
What counts as validity evidence? Examples and prevalence in a systematic review of simulation-based assessment	Cook	Advances in Health Sciences Education	2014	USA	Systematic review
When I say. . . Validity	Cook	Medical Education	2014	USA	When I Say…
A contemporary approach to validity arguments: a practical guide to Kane's framework	Cook	Medical Education	2015	USA	Cross-cutting edge
Much ado about differences: why expert-novice comparisons add little to the validity argument	Cook	Advances in Health Sciences Education	2015	USA	Perspective
The trouble with validity: what is part of it and what is not	Knorr	Medical Education	2015	Germany	Commentary

Evolving conceptualisations of validity: impact on the process and outcome of assessment	St Onge	Medical Education	2015	Canada	Commentary
Consequences Validity Evidence: Evaluating the Impact of Educational Assessments	Cook	Academic Medicine	2016	USA	General article
Validation of educational assessments: a primer for simulation and beyond	Cook	Advances in Simulation	2016	USA	Methodology article
When assessment data are words: validity evidence for qualitative educational assessments	Cook	Academic Medicine	2016	USA	General article
‘‘Face validity’’ is not a legitimate type of validity evidence!	Royal	American Journal of Surgery	2016	USA	Editorial opinion
Validity: one word with a plurality of meanings	St Onge	Advances in Health Sciences Education	2016	Canada	Research
Validation of learning assessments: a primer	Peeters	Currents in Pharmacy Teaching & Learning	2017	USA	Methodology Matters
Four tenets of modern validity theory for medical education assessment and evaluation	Royal	Advances in Medical Education and Practice	2017	USA	Perspective
The foundations of measurement and assessment in medical education	Takavol	Medical Teacher	2017	United Kingdom	AMEE guide
Surgical Education, Simulation, and Simulators—Updating the Concept of Validity	Goldenberg	Current Urology Reports	2018	Canada	Review

Validity as a social imperative for assessment in health professions education: a concept analysis	Marceau	Medical Education	2018	Canada	Research
Competency-based training and simulation: making a “valid” argument	Noureldin	Journal of Endourology	2018	USA, Egypt	Review
Applying Kane’s validity framework to a simulation based assessment of clinical competence	Tavares	Advances in Health Sciences Education	2018	Canada	Research
Assuring the quality of programmatic assessment: Moving beyond psychometrics	Uijtdehaage	Perspectives on Medical Education	2018	USA	Commentary
When I say. . . Validity argument	von Bergmann	Medical Education	2018	Canada	When I Say…
Collecting Validity Evidence: A Hands-on Workshop for Medical Education Assessment Instruments	Paul	MedEdPortal	2019	USA	Workshop
The compatibility principle: on philosophies in the assessment of clinical competence	Tavares	Advances in Health Sciences Education	2019	Canada	Reflections
A history of assessment in medical education	Schuwirth	Advances in Health Sciences Education	2020	Australia	Invited paper
The validity argument: Addressing the misconceptions	Gasmalla	Medical Teacher	2021	Sudan	Personal view
Convince Me! How a Well-Constructed Validity Argument Supports the Decisions	Adler	Journal of Pediatrics	2022	Canada	Editorial

We Make About Learners					

Articles are listed in chronological order. Journal-specific article type labels (e.g. When I say…) were used when available. Author affiliations were derived from those listed in each publication and are not intended to reflect the authors' countries of origins.
